# Spatial Bayesian Modeling Applied to the Surveys of *Xylella fastidiosa* in Alicante (Spain) and Apulia (Italy)

**DOI:** 10.3389/fpls.2020.01204

**Published:** 2020-08-14

**Authors:** Martina Cendoya, Joaquín Martínez-Minaya, Vicente Dalmau, Amparo Ferrer, Maria Saponari, David Conesa, Antonio López-Quílez, Antonio Vicent

**Affiliations:** ^1^Centre de Protecció Vegetai i Biotecnología, Institut Valencià d’Investigacions Agràries (IVIA), Moncada, Spain; ^2^Data Science Area, Basque Center for Applied Mathematics (BCAM), Bilbao, Spain; ^3^Servei de Sanitat Vegetal, Conselleria d’Agricultura, Desenvolupament Rural, Emergència Climàtica i Transició Ecológica, Silla, Spain; ^4^Instituto per la Protezione Sostenibile delle Piante, Sede Secondaria di Bari Consiglio Nazionale delle Ricerche (CNR), Bari, Italy; ^5^Departament d’Estadística i Investigació Operativa, Universitat de València, Burjassot, Spain

**Keywords:** hierarchical Bayesian models, integrated nested Laplace approximation, stochastic partial differential equation, *Xylella fastidiosa*, species distribution models, olive quick decline, almond leaf scorch

## Abstract

The plant-pathogenic bacterium *Xylella fastidiosa* was first reported in Europe in 2013, in the province of Lecce, Italy, where extensive areas were affected by the olive quick decline syndrome, caused by the subsp. *pauca*. In Alicante, Spain, almond leaf scorch, caused by *X. fastidiosa* subsp. *multiplex*, was detected in 2017. The effects of climatic and spatial factors on the geographic distribution of *X. fastidiosa* in these two infested regions in Europe were studied. The presence/absence data of *X. fastidiosa* in the official surveys were analyzed using Bayesian hierarchical models through the integrated nested Laplace approximation (INLA) methodology. Climatic covariates were obtained from the WorldClim v.2 database. A categorical variable was also included according to Purcell’s minimum winter temperature thresholds for the risk of occurrence of Pierce’s disease of grapevine, caused by *X. fastidiosa* subsp. *fastidiosa*. In Alicante, data were presented aggregated on a 1 km grid (lattice data), where the spatial effect was included in the model through a conditional autoregressive structure. In Lecce, data were observed at continuous locations occurring within a defined spatial domain (geostatistical data). Therefore, the spatial effect was included *via* the stochastic partial differential equation approach. In Alicante, the pathogen was detected in all four of Purcell’s categories, illustrating the environmental plasticity of the subsp. *multiplex*. Here, none of the climatic covariates were retained in the selected model. Only two of Purcell’s categories were represented in Lecce. The mean diurnal range (*bio2*) and the mean temperature of the wettest quarter (*bio8*) were retained in the selected model, with a negative relationship with the presence of the pathogen. However, this may be due to the heterogeneous sampling distribution having a confounding effect with the climatic covariates. In both regions, the spatial structure had a strong influence on the models, but not the climatic covariates. Therefore, pathogen distribution was largely defined by the spatial relationship between geographic locations. This substantial contribution of the spatial effect in the models might indicate that the current extent of *X. fastidiosa* in the study regions had arisen from a single focus or from several foci, which have been coalesced.

## Introduction

*Xylella fastidiosa* (Wells et al., 1987) is a gram-negative plant-pathogenic bacterium of the Xanthomonadaceae family. It colonizes the xylem tissues of a wide range of plant species: The latest update of the host plants database includes 595 species belonging to 85 different families (EFSA, 2020). However, it is only in some combinations of host plant and bacterial strain that infections can result in some of the most destructive diseases, such as Pierce**’**s disease (PD) of grapevine and olive quick decline syndrome (OQDS), threatening several crops of great economic importance. Hence, to preserve the European Union (EU) territory from its introduction from areas where the presence of the bacterium is known, *X. fastidiosa* is regulated as a quarantine organism by Regulation (EU) 2016/2031 and Implementing Regulation (EU) 2019/2072. Recently, it has been included in the list of the priority pests for the EU (Regulation 2019/1702).

A total of six subspecies of *X. fastidiosa* have been proposed to date, although based on genetic differences, host ranges, and associated diseases, the majority of the bacterial strains fall into four main subspecies. *X. fastidiosa* subsp. *fastidiosa* causes, among others, PD and almond leaf scorch; *X. fastidiosa* subsp. *pauca* has been identified as the cause of citrus variegated chlorosis (CVC), leaf scorch of coffee, and OQDS. *X. fastidiosa* subsp. *multiplex* has been found in about 140 plant species and is associated with leaf scorch diseases in numerous tree species, including almond (EFSA, 2020). *X. fastidiosa* subsp. *sandyi* has been associated with oleander leaf scorch (Schaad et al., 2004; Janse and Obradovic, 2010). Nevertheless, the Committee on the Taxonomy of Plant Pathogenic Bacteria of the International Society of Plant Pathology (ISPP) only considers as valid subspecies names *fastidiosa* and *multiplex* (Bull et al., 2012). In addition to these, the subsp. *tashke* (Randall et al., 2009) and subsp. *morus* (Nunney et al., 2014b) have been proposed. Indeed, recently, the *X. fastidiosa* strain isolated from *Pyrus pyrifolia* in Taiwan has been proposed to form a distinct species in the genus *Xylella*: the *X. taiwanensis* sp. nov (Su et al., 2016).

Xylem sap-feeding insects represent the only natural means of spreading *X. fastidiosa* between plants. The bacterium colonize the foregut of the insects that act as vectors. The human-assisted spread of *X. fastidiosa*, however, is mainly through the movement of infected plants and grafting. Therefore, two habitats are distinguished in the life cycle of *X. fastidiosa*, one is the foregut of the insect vectors, and the second is the xylem tissue of the host plant (Almeida and Nunney, 2015). All confirmed vectors of *X. fastidiosa* are hemiptera belonging to two groups of insects: sharpshooters (Cicadellidae family and Cicadellinae subfamily) (Almeida and Purcell, 2003) and spittlebugs (Aphrophoridae, Cercopidae, and Clastopteridae families) (Cornara et al., 2018). The transmission efficiency of *X. fastidiosa* by vectors depends on several factors, such as the ecology and abundance of the insects present in the area and the population density of the bacterium in the host plants (Almeida et al., 2005).

In the American continent, species of sharpshooters are the main vectors of *X. fastidiosa*. Most of the available information on these vectors is based on studies conducted with *Homalodisca vitripennis* (Germar) (Hemiptera: Cicadellidae), also known as *Homalodisca coagulata* (Say). It is an invasive species and currently considered the main vector of PD in California, US. In Europe, sharpshooters are not abundant or widespread. Different species of spittlebugs have been found to play, or potentially play, a major role in the transmission of *X. fastidiosa* (Cornara et al., 2018). In particular, *Philaenus spumarius* L. (Hemiptera: Aphrophoridae) has been considered the main vector of *X. fastidiosa* subsp. *pauca* in Italy (Cavalieri et al., 2019). This species of spittlebug is widely distributed and is characterized by its color polymorphism and the secretion of foam by nymphs.

*Xylella fastidiosa* was first described as the cause of PD in California. For decades, the geographic distribution of *X. fastidiosa* was restricted to the American continent, but in 2013, it was first reported in Europe as causing OQDS in southern Italy, with thousands of hectares infected and millions of trees killed. Two years later, *X. fastidiosa* subsp. *multiplex* was reported in Corsica and Provence-Alpes-Côte d’Azur in France, where, in this latter case, *X. fastidiosa* subsp. *pauca* was also recently detected (DG SANTE, 2020). Three subspecies of *X. fastidiosa* (i.e., *multiplex*, *pauca*, and *fastidiosa*) were reported in the Balearic Islands. Outbreaks associated with *X. fastidiosa* subsp. *multiplex* were detected in Alicante and Madrid, Spain, Porto District, Portugal, and Tuscany, Italy. In 2019, the presence of *X. fastidiosa* subsp. *fastidiosa* was officially reported in Israel (EPPO, 2019a). After the first detection of *X. fastidiosa* in the EU, emergency phytosanitary measures were laid down under Decision (EU) 2015/789 in all EU Member States with the aim of preventing further introduction and spread within the EU territory.

The current geographical distribution of *X. fastidiosa* comprises areas with different climate types (EFSA, 2019). Although the highest prevalence of the pathogen occurs in tropical and subtropical climates, it is also found in regions that are much colder and/or drier. Despite the relatively wide temperature range where *X. fastidiosa* develops, it should be noted that its performance at low temperatures depends largely on the interaction between the subspecies and the host plant. This would explain the differences in the geographical range and prevalence of the different *X. fastidiosa* subspecies on their associated hosts. For instance, in California, US, the severity of almond leaf scorch is much lower than that of Pierce’s disease in grapevines, although they are caused by the same subspecies of *X. fastidiosa* (Purcell, 1997). Similarly, in North America, *X. fastidiosa* subsp. *multiplex* is present in areas that are colder than those where *X. fastidiosa* subsp. *fastidiosa* is prevalent (EFSA, 2015). It is not known whether the geographic distribution of the different *X. fastidiosa* subspecies is associated with their ability to thrive at low temperatures, to the distribution and abundance of their host plants and vectors, or simply because they have not reached their maximum geographical and/or environmental extent.

In this context, species distribution models (SDMs) can be a useful tool to study the geographic range of *X. fastidiosa*, given that they link spatial occurrence data with multivariate environmental data that can be used to estimate the relationship between the species and its habitat and subsequently predict spatial occurrence in unsampled locations or time periods (Martínez-Minaya et al., 2018a). SDMs have been used in previous works in order to describe the relationship between *X. fastidiosa* and climatic variables. Bosso et al. (2016) used the Maxent model to estimate the potential distribution of *X. fastidiosa* in the Mediterranean Basin based on climatic variables, including climate change scenarios. Godefroid et al. (2019) analyzed the potential effect of climate change on different subspecies of *X. fastidiosa*. The SDMs Bioclim and Domain were fitted using presence data of the subspecies *multiplex* and *pauca* to estimate their potential geographic distribution under current and future climate conditions. Furthermore, the severity of PD and its relationships with climatic variables were modeled by means of ordinal regression using the PD risk maps proposed by Purcell (Anas et al., 2008). Hernández and García (2019) used ecological niche models to estimate the potential distribution of *X. fastidiosa* on a global scale. EFSA, 2019 used SDM ensemble modeling to asses the potential establishment of *X. fastidiosa* in the EU. All these previous studies with SDMs for *X. fastidiosa* have two characteristics in common: They used presence-only data or generated pseudo-absences such as Maxent and did not include spatial autocorrelation. Models based on presence-only data are indeed a useful tool when absence data are not available (Franklin, 2010). However, without the absence data, the accuracy of the models can be overestimated (Brotons et al., 2004). In addition, when the spatial dependence of the data is ignored, the degree of uncertainty can be underestimated, generating imprecise estimations of the parameters and providing relatively low predictive capacity (Latimer et al., 2006; Martínez-Minaya et al., 2018b).

In this study, the geographic distribution of *X. fastidiosa* was analyzed in two affected regions in Europe: Alicante (Spain) and the province of Lecce in the Salento peninsula (Apulia, Italy). These two study regions were selected due to them having different but relatively simple scenarios with regard to the prevalent subspecies of *X. fastidiosa* and the main hosts affected. In Alicante, only *X. fastidiosa* subsp. *multiplex* ST6 was identified, mainly affecting almonds (Landa et al., 2020). On the other hand, in Apulia, only *X. fastidiosa* subsp. *pauca* ST53 was identified, mainly affecting olives (Saponari et al., 2013). The presence/absence data of *X. fastidiosa* were analyzed in each study region with Bayesian hierarchical models, which allowed us to include different spatial dependencies of each dataset. Furthermore, in Bayesian statistics observations and parameters are considered as random variables and so their uncertainty can be incorporated in a natural way *via* Bayesian hierarchical models (Banerjee et al., 2004). Computational advances have made it possible to approximate the posterior distribution of the parameters involving these complex models by means of integrated nested Laplace approximation (INLA) (Rue et al., 2009). The primary scope of this study was to determine the influence of climatic variables on the geographic distribution of the pathogen, as well as the spatial relationship between the positive locations in each region. Results in the form of risk maps will help Plant Health Authorities to optimize the official delimiting surveys for *X. fastidiosa* as well as to implement control strategies, such as eradication or containment, as established by the Decision (EU) 2015/789.

## Materials and Methods

### Databases

**Figure 1:**
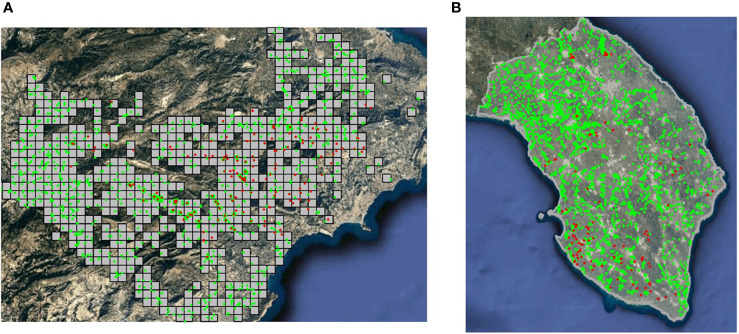
Presence (•) and absence (•) of *X. fastidiosa*. **(A)** Sampled grid cells (1 km^2^) in the demarcated area in Alicante, Spain, in 2017; **(B)** sampling in the province of Lecce, Italy, during the 2013–2014 campaign.

As the kind of spatial data gathered defines the final hierarchical spatial model used, it is necessary to describe in detail the two different databases available. A georeferenced dataset was provided by the Plant Health Authority (*Sanitat Vegetal*) of the *Generalitat Valenciana*, including the results of the official delimiting surveys for *X. fastidiosa* in 2017 in the demarcated area in the province of Alicante, Spain. Surveillance (i.e., inspection and sampling) in the demarcated area was based on the specifications established by Decision (EU) 2015/789, according to which, a visual examination of the plants specified as susceptible and, in case of suspicion of infection by the pathogen, the collection of samples and laboratory testing. A total of 3203 samples were considered, 206 of them were positive (i.e., presence) for *X. fastidiosa*, and 2997 were negative (i.e., absence) based on real-time PCR (EPPO, 2019b) (**Figure 1A**). Only *X. fastidiosa* subsp. *multiplex* ST6 was detected. Samples were taken from 57 different plant species, but all the positives were in almond (*Prunus dulcis*). Nevertheless, all sampled plant species were included in the analysis, as they were considered susceptible to *X. fastidiosa* (EFSA, 2020). The total number of samples was presented aggregated on the 1 km × 1 km spatial grid used by *Sanitat Vegetal*. Non-sampled grid cells were removed, since most of them corresponded to mountain peaks with difficult access and/or the absence of host plants; thus, the study area had an extension of 638 km^2^.

Data on the distribution of *X. fastidiosa* in Apulia (Italy) were obtained from the official surveillance program conducted by the Plant Health Authority of the *Regione Puglia* from 2013 to 2018. Due to the different surveillance strategies each year, only data from the province of Lecce (2,766 km^2^) during the first sampling campaign (from November 2013 to December 2014) were considered for further analysis. Samples were first tested by enzyme-linked immunosorbent assay (ELISA), and all samples yielding positive reactions were confirmed by a second round of assay using real-time PCR (EPPO, 2019b). The selected dataset included a total of 4,205 samples, 224 of them were positive (i.e., presence) for *X. fastidiosa* and 3,981 were negative (i.e., absence) (**Figure 1B**). Only *X. fastidiosa* subsp. *pauca* ST53 was detected. In this case, data were not presented aggregated on a grid structure as before. As a result, samples were considered as georeferenced observations in a continuous space.

Climatic data for the demarcated area in Alicante and the province of Lecce were obtained from the WorldClim v.2 database with a resolution of 30” arc sec (Fick and Hijmans, 2017). This database contains monthly average data of temperature and precipitation from 1970 to 2000 and 19 derived bioclimatic variables. In addition, accumulated degree days (*T_base_*,15°C) during the vegetative growth period of the main host species in each region were also considered, i.e., from February to October for almond in Alicante (Pou, 2004) and from April to October in olive in Lecce (Rallo and Cuevas, 2017). The UTM coordinate system was used in all the spatial datasets with the raster package for R software (Hijmans and van Etten, 2012; R Core Team, 2019).

For Alicante, the climatic variables considered relevant by Martinetti and Soubeyrand (2019) for explaining the presence of *X. fastidiosa* in areas of France where subsp. *multiplex* is prevalent were also included in the analysis. These variables were the average minimum temperature in winter from December to March (*tmin*), the average precipitation during the dry season from July and August (*precd*) and the solar radiation (*srad*).

Purcell’s classification, based on the minimum winter temperature was proposed in the US to estimate the risk of PD (Anas et al., 2008). This classification consists of four categories: severe (>4.5°C), moderate (1.7 to 4.5°C), occasional (-1.1 to 1.7°C), and negligible (<-1.1°C). A categorical variable considering these four categories based on the minimum temperature of the coldest month (*bio6*) in WorldClim v.2 was included in the analysis in both study regions.

Due to the nature of the climatic variables, high linear correlations were found among most of them (**Figure S1**). In order to minimize potential problems of multicollinearity, a variable selection was performed based on the Pearson’s correlation coefficient excluding pairs of variables with |*r*| > 0.7 (Dormann et al., 2013). Alternatively, principal component analysis (PCA) was conducted to reduce the number of variables and obtain new uncorrelated variables. Since the climatic variables had different metrics, PCA was performed based on the correlation matrix. The correlation of each variable with the principal components (PCs) was expressed by a rotation with the Varimax method. The variable temperature annual range (*bio7*) was excluded from PCA because it is a linear combination of the variables maximum temperature of the warmest month (*bio5*) and minimum temperature of the coldest month (*bio6*).

### Models

Two different Bayesian hierarchical spatial models were used to analyze the variation of the presence of *X. fastidiosa* in the study areas. Bayesian hierarchical models allow the incorporation of sources of variability and non-observed uncertainty. Nevertheless, computational methods, such as Markov chain Monte Carlo (MCMC) and INLA are generally required in order to obtain posterior distributions of the parameters and hyperparameters. In particular, the INLA methodology (Rue et al., 2009) is designed for latent Gaussian models (LGMs), a large class of models including the hierarchical spatial models used here, and provides accurate results in shorter computing times compared with MCMC (Blangiardo and Cameletti, 2015).

LGMs can also be considered as a particular case of the structured additive regression models (STAR), (Fahrmeir et al., 2013), where the mean of the response variable *Y_t_* is linked to a structured predictor that accounts the various effects in an additive way:

(1) $$\eta_{i}=g(\mu_{i})=\beta_{0}+\sum_{j=1}^{N_{\beta }}\beta_{j}x_{ji}+\sum_{k=1}^{N_{f}}f_{k}(z_{ki})+v_{i},\;i=1,\ldots ,\;n,$$

where *η_i_* enters the likelihood through a link function, *β_0_* is the intercept of the model, *β_j_* are the fixed effects of the model, *f_k_* denote any smooth effects, and *v_i_* represents any random effect, such as a spatial effect. The models which we deal with in this work include only fixed effects and in some cases a structured spatial term. The prior knowledge of the additive predictor is expressed using Gaussian prior distributions. In this context, all the latent Gaussian variables can be seen as components of a vector known as the latent Gaussian field. This class of models have several applications due to their flexibility and can be fitted using the INLA methodology with the R package R-INLA.

In both case studies, logistic regressions were performed. In the case of Alicante, as we considered the total number of positives of the total number of samples in each grid cell, the response variable (*y_i_*) was assumed to be a Binomial distribution, i.e., *y_i_* ~ Binomial (*n_i_, π_i_*), where *π_i_* is the probability of a sample being positive in the grid cell *i*, and *n_i_* is the total number of samples in that grid cell. On the other hand, the response variable *y_i_* in Lecce was assumed to follow a Bernoulli distribution, where 0 indicates the absence and 1 the presence of *X. fastidiosa* at location *i*, that is, *y_i_ ~* Bernoulli (*π_i_)*, *π_i_* being the probability of presence at location *i*. But as the samples were not collected in the same way, different structures for the spatial random effect were employed in each case.

#### Model for Alicante

Data in Alicante come from a sampling carried out in a georeferenced regular lattice. A common way to deal with the spatial dependence amongst the grid cells is to consider an intrisic Gaussian Markov random field (GMRF) model, also known as the Besag model presented by Besag (1974). The main idea is to construct a model where each random effect *v_i_* conditionally to its neighbor random effects has a Gaussian distribution with a mean equal to the average of the neighbors and a precision proportional to the number of neighbors:

(2) $$v_{i}|v_{-i}∼N\left(\frac{1}{k_{i}}\sum_{i∼j}v_{j},\frac{1}{\tau_{v}k_{i}}\right),\;i\neq j,$$

where *τ_v_* is the precision of the random effect and *k_i_* the number of neighbors corresponding to the grid cell *i*, which has a set of neighbors *j*≠*i*. As some grid cells did not have any adjacent ones, the neighborhood relation was established at a distance of 2.5 km, that is, two grid cells were considered neighbors if the distance between their centroids was ≤2.5 km. Due to the 1 km × 1 km resolution of the data, 2.5 km was the minimum distance resulting in at least one neighbor for all grid cells. However, this kind of effect only accounts for similarities between grid cells, and it does not take into account the individual variability of each grid cell. Then, the Besag, York and Mollié model (Besag et al., 1991) was used, simply adding an independent random effect to the model. In this way, *η_i_ = β_0_+**X**_i _**β**+v_i_+u_i_*, where *β_0_* is the intercept, **β** represents the effect of the covariates ***X***_i_, *v_i_* is the spatial effect, and *u_i_* is the independent random Gaussian effect. Simpson et al. (2017) proposed a reparameterisation of this model that allows an straightforward incorporation of penalized complexity priors (PC-prior) (Riebler et al., 2016; Simpson et al., 2017) to the model hyperparameters, including a standardized spatial component v*:

(3) $$\eta_{i}=\beta_{0}+X_{i}\beta +\frac{1}{\tau }\left(\sqrt{1-\phi }u_{i}+\sqrt{\phi }v_{i}^{*}\right),$$

where τ controls the marginal variance of v* and ***u***. In addition, it incorporates the mixing parameter 0 ≤ ϕ ≤ 1, which measures the proportion of variance explained by v*, so values close to 1 would imply a strong weight of the spatial component, while ϕ = 0 only that of the independent random effect.

Consequently, the complete model with covariates, random effects and all the necessary prior distributions for the parameters and hyperparameters involved had the following structure:

(4)             yi∼Binomial(ni, πi), i=1, … , 638,logit(πi)=β0+Xiβ+1τ(1−ϕui+ϕvi*),P(β0)∝1,          βm∼N(μ=0, τ=10−3), m=1, … ,Nβ            τ∼PC-prior(0.5/0.31,0.01),            ϕ∼PC-prior(0.5,2/3),

where *n_i_* is the total number of samples for each cell *i*, *π_i_* is the probability that a sample taken in cell *i* is positive, and **β** is the vector of coefficients of covariates ***X**_i_*, to which a non-informative prior has been assigned in the form of a Normal distribution with mean 0 and precision of 10^-3^. The scaled spatial effect $v_{i}^{*}$ is specified by an intrinsic conditional autoregressive (ICAR) distribution, and *u_i_* is the independent random effect, where *u_i_~N*(*0,**I***). Following Simpson et al. (2017), a PC-prior for the precision τ was defined as $P\left(1/\sqrt{\tau }>0.5/0.31\right)=0.01$. On the other hand, a prior distribution was assigned for the mixing parameter ϕ that assumes that the independent random effect explaining more variability than the spatial component, where *P*(ϕ < 0.5) = 2/3.

#### Model for Lecce

Another Bayesian hierarchical spatial model, more specifically a Bayesian geostatistical model, was used to analyze the presence of *X. fastidiosa* in Lecce. As mentioned above, this was based on the fact that data were not presented aggregated on a grid structure as in the other dataset, therefore samples were considered as georeferenced observations in a continuous space. In this case, the spatial dependence is expressed *via* the spatial effect ***w*** (geostatistical term) that is assumed to follow a multivariate Gaussian distribution whose covariance matrix $\sigma_{w}^{2}H(\phi )$ depends on the distance between locations, and the hyperparameters $\sigma_{w}^{2}$ and ϕ, the variance, and the range of the spatial effect, respectively. In order to fit and predict using this kind of model, where an indexed continuous Gaussian field (GF) is included in the formula, Lindgren et al. (2011) proposed an explicit link between GMRF and GF with a Matérn covariance structure *via* a weak solution to a stochastic partial differential equation (SPDE). With this approximation, the spatial term is reparametrized as $w∼N(0,\;Q^{-1}(\kappa ,\;\tau ))$, depending on two different parameters, κ and τ. More precisely, the range is approximately $\phi =\sqrt{\frac{8}{\kappa }}$ and the variance is $\sigma_{w}^{2}=\frac{1}{4\pi \kappa^{2}\tau^{2}}$ Lindgren et al. (2011). However, instead of using the default parametrization, Krainski et al. (2019) recommended using the one that is more intuitive to control the parameters through the marginal standard deviation and the range.

Lastly, we specified prior distributions for the parameters and hyperparameters. In particular, Normal vague priors with mean and precision were used for the regression coefficients. Following Fuglstad et al. (2019), PC-priors were used for the range and the standard deviation of the spatial field.

Taking all this into account, the final model with covariates, random effects, and all the necessary prior distributions for the parameters and hyperparameters involved had the following structure:

(5) $$\begin{array}{lll} \;y_{i} & ∼ & \text{Bernoulli}(\pi_{i}),\;i=1,\;\ldots \;,\;4205,\\ \text{logit}(\pi_{i}) & = & X_{i}\beta +w_{i},\\ \;w & ∼ & N(0,\;Q^{-1}(\phi ,\sigma_{w})),\\ P(\beta_{0}) & \propto & 1,\\ \;\beta_{m} & ∼ & N(\mu =0,\tau =10^{-3}),\;m=1,\;\ldots \;,\;N_{\beta }\\ \;\phi & ∼ & \text{PC-prior}(\mu_{\phi },\;0.5),\\ \;\sigma_{w} & ∼ & \text{PC-prior}(1,\;0.5), \end{array}$$

where *π_i_* is the probability of the presence of the pathogen in the location *i* and **β** is the vector of the coefficients of the covariates ***X**_i_* and ***w*** is the spatial effect. The PC-priors were defined as P(*ϕ* < *μ_ϕ_*= 0.5) and *P*(σ>1)=0.5 for the range and standard deviation, respectively, where *μ_ϕ_* was chosen as 50% of the diameter of the geographic region under study (Fuglstad et al., 2019).

### Model Selection

Given the large number of models resulting from all the possible combinations of covariates, model selection was carried out. As indicated, Pearson’s correlation coefficients among covariates were previously calculated to assist in variable selection and minimize potential problems of multicollinearity. If the correlation between two variables was greater than 0.7, one of those covariates was taken out of the analysis (Dormann et al., 2013). With the resulting covariates, all possible 2*^k^* (where *k* represents the number of components of the model: covariates and the random effects) models were evaluated and the best one was chosen according to information criteria (Heinze et al., 2018). In this work, we used the Watanabe Akaike information criterion (WAIC) (Watanabe, 2010; Gelman et al., 2014), which is the sum of two components, one quantifying model fit and other evaluating model complexity. The predictive capacity of the models was evaluated by cross validation using the logarithmic conditional predictive ordinate (LCPO) (Pettit, 1990; Roos and Held, 2011). Models with the lowest values of WAIC and LCPO were selected. When several models presented similar information criteria, the parsimony criterion was applied and models with fewer covariates were selected.

## Results

### Alicante

All the 23 climatic variables included in the analysis showed high linear correlation (**Figure S1A**). Nevertheless, annual mean temperature (*bio1*), temperature annual range (*bio7*), and precipitation of the wettest month (*bio13*) had *r* < |0.7|. These covariates presented low variability in the study area, minimum and maximum values of *bio1* were 10.49 and 17.69°C, respectively. The covariate *bio7* varied between 24.70 and 30.93°C, and *bio13* between 46 and 67.31 mm. Furthermore, the distribution of *X. fastidiosa* in the study area was not coincident with those covariates (**Figure S2**). Despite the relative climatic homogeneity, the four categories defined by Purcell based on the minimum winter temperature were represented in the study area. Nevertheless, *X. fastidiosa* was detected in all of them. Moreover, the proportion of positive samples in the area for each category was similar with 1.78% in the severe category (>4.5°C), 8.28% in the moderate category (1.7 to 4.5°C), 6.03% in the occasional category (-1.1 to 1.7°C), and 2.29% in the negligible category (<-1.1°C) (**Figure S2D**).

All model combinations (n = 32) with the three selected climatic covariates (*bio1*, *bio7*, and *bio13*), Purcell’s categories, and the spatial effect were fitted. The model with the lowest WAIC value was the one including only the spatial effect, with a WAIC of 617.627 and an LCPO of 1.638 (**Table S1**).

**Figure 2:**
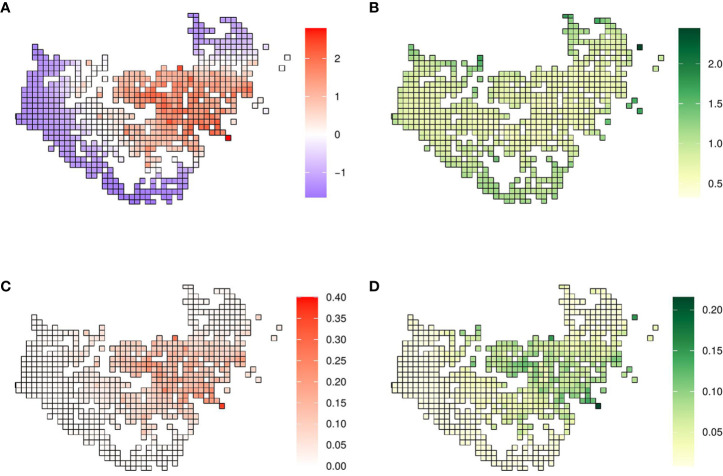
Model with the spatial effect. **(A)** Mean and **(B)** standard deviation of the posterior distribution of the spatial effect. **(C)** Mean and **(D)** standard deviation of the posterior predictive distribution of the probability of *X. fastidiosa* presence in the demarcated area in Alicante, Spain.

The posterior distribution of the spatial effect was in the linear scale, meaning that positive values imply a higher probability of the presence of *X. fastidiosa* and negative values were associated with a lower probability, and it was related to the data according to the neighborhood structure defined. The posterior mean of the spatial effect took positive values in areas where there was a high proportion of positives, while negative values were concentrated where *X. fastidiosa* was not detected (**Figure 2A**). The standard deviation of the posterior distribution of the spatial effect varied between 0.32 and 2.44, with lower uncertainty in areas where the spatial effect was greater, and the highest values in the isolated cells (**Figure 2B**). The posterior mean of the mixing parameter ϕ was 0.931, indicating that most of the variability was explained by the spatial effect (**Table 1**).

**Table 1:** Mean, standard deviation (sd), quantiles (*Q*), and mode for the parameters and hyperparameters (τ, ϕ) of the best model for the distribution of *X. fastidiosa* in the demarcated area in Alicante, Spain.

Parameters	Mean	sd	Q_0.025_	Q_0.5_	Q_0.975_	Mode
*β*_0_	-3.524	0.173	-3.884	-3.516	-3.208	-3.500
**Hyperparameters**	**Mean**	**sd**	**Q_0.025_**	**Q_0.5_**	**Q_0.975_**	**Mode**
τ	0.936	0.264	0.529	0.898	1.557	0.827
ϕ	0.931	0.063	0.763	0.949	0.996	0.989

β_0_ is the intercept, τ is the precision of the spatial effect and ϕ the mixing parameter.

The mean of the predictive posterior distribution of the response variable was expressed in terms of probability (0–1), in our case with values ranging from 0.01 to 0.36. The highest probability of the presence of *X. fastidiosa* was in grid cells where the posterior distribution of the spatial effect was also highest. In grid cells where the pathogen was not detected nor in their neighboring cells, the probability of presence was close to zero (**Figure 2C**). The standard deviation of the predictive posterior distribution increased with the probability of presence, with the highest value of 0.22 (**Figure 2D**).

PCA was performed with the original covariates, resulting in 96.4% explained variance with the first three PCs (**Table S2**). The covariates mean temperature of coldest quarter (*bio11*) and precipitation seasonality (*bio15*) had a strong influence on PC1, with positive coefficients greater than 0.97. PC2 was mainly defined by the precipitation in the wettest month (*bio13*), which had a coefficient of 0.94. In PC2, the covariates related with temperature had negative coefficients, whereas other covariates of precipitation were positive. In PC3, the covariate with the highest weight (0.97) was the mean diurnal range (*bio2*). Nevertheless, when plotting the PCs there was no clear coincidence with the distribution of *X. fastidiosa* (**Figure S3**). All model combinations were fitted (n = 32) considering the three PCs, Purcell’s categories, and the spatial effect. Again, the model including only the spatial effect was selected based on WAIC and LCPO values, and it was also found to be the most parsimonious (**Table S1**).

### Province of Lecce

All the 20 climatic variables included in the analysis showed high linear correlation (**Figure S1B**). Among them, annual mean temperature (*bio1*), mean diurnal range (*bio2*), mean temperature of the wettest quarter (*bio8*), mean temperature of the driest quarter (*bio9*), annual precipitation (*bio12*), and precipitation of the driest month (*bio14*) had *r*<|0.7|. Most of the positives were observed in the southwestern area of Lecce, coinciding with low values of *bio2*, *bio9*, *bio12*, and *bio14*, and high values of *bio1* and *bio8* (**Figure S4**). Some positives were observed in the eastern part of the province, where *bio8* and *bio14* had the lowest values and *bio12* displayed the highest. Climatic variables had low variability, for instance, with *bio1* ranging from 16.07 to 17.23°C and *bio12* from 506.6 to 679.7 mm. With regard to Purcell’s categories based on minimum winter temperatures, only two (moderate and severe) were represented in the province of Lecce (**Figure S4G**). Hence, this categorical variable was not further considered in the models.

All model combinations (n = 128) with the selected climatic covariates (*bio1*, *bio2*, *bio8*, *bio9*, *bio12*, and *bio14*), and the spatial effect were fitted. The model selected was the one including the covariates *bio2* and *bio8* and the spatial effect. This model was chosen because it was the most parsimonious of those with the lowest values of WAIC and LCPO, meaning good model fit and predictive capacity with fewer covariates (**Table S3**).

**Figure 3:**
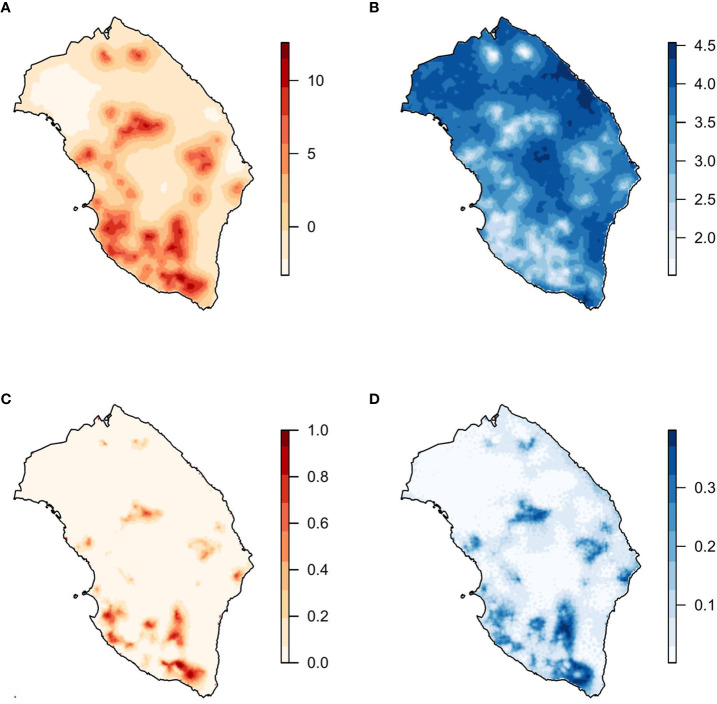
Model with the covariates mean diurnal range (*bio2*), temperature of the wettest quarter (*bio8*), and the spatial effect. **(A)** Mean and **(B)** standard deviation of the posterior distribution of the spatial effect. **(C)** Mean and **(D)** standard deviation of the posterior predictive distribution of the probability of *X. fastidiosa* presence in Lecce, Italy.

In the selected model, the posterior mean of the parameters of *bio2* and *bio8* was negative (**Table 2**). Regarding the spatial effect, positive values of the mean posterior distribution were associated with a higher probability of the presence of *X. fastidiosa*, while negative values were related to a lower probability. The mean of the posterior distribution of the spatial effect had higher values and lower variability in the areas where *X. fastidiosa* was first detected on the southwestern coast (**Figure 3**). The posterior predictive distribution of the response variable showed that the model was strongly influenced by the spatial effect. Consequently, the probability of the presence of *X. fastidiosa* was much higher in the areas around positive findings, along with higher values of the spatial effect, but practically null in the areas farther away from the positives (**Figure 3**). In addition, the standard deviation (uncertainty) of the predictive posterior distribution increased with the probability of presence.

**Table 2:** Mean, standard deviation (sd), quantiles (*Q*), and mode for the parameters and hyperparameters (ϕ, *σ_w_*) of the best model for the distribution of *X. fastidiosa* in Lecce, Italy, based on mean diurnal range (*bio2*) and mean temperature of wettest quarter (*bio8*).

Parameters	Mean	sd	*Q*_0.025_	*Q*_0.5_	*Q*_0.975_	Mode
*β*_0_	17.465	11.509	-4.153	17.087	41.300	16.413
*bio*2	-1.149	0.744	-2.639	-1.144	0.313	-1.133
*bio*8	-1.216	0.706	-2.727	-1.177	0.064	-1.107
**Hyperparameters**	**Mean**	**sd**	***Q*_0.025_**	***Q*_0.5_**	***Q*_0.975_**	**Mode**
ϕ	5609.527	1112.976	3858.475	5455.545	8202.005	5132.501
*σ_w_*	4.837	0.753	3.527	4.778	6.480	4.662

β_0_ is the intercept and bio2 and bio8 represent the parameters of the covariates mean diurnal range and mean temperature of wettest quarter, respectively. ϕ and σ_w_ are the range and variance of the spatial effect, respectively.

The three PCs with climatic covariates explained an accumulated variance of 87.7% (**Table S2**). PC1 was strongly influenced by two covariates: the mean diurnal range (*bio2*) with a positive coefficient of 0.96 and minimum temperature of the coldest month (*bio6*) with a negative coefficient of -0.96. The precipitation of the wettest month (*bio13*), the wettest quarter (*bio16*), and the coldest quarter (*bio19*) were the covariates that made a greater contribution in PC2, all of them with positive coefficients higher than 0.95. PC3 was mainly driven by the accumulated degree days over 15°C (*ADD*), with a coefficient of 0.97. The Ionian coast in Lecce, where most of the positives were located, showed the lowest values of PC1, although *X. fastidiosa* was also found in areas with high values for PC1. Similarly, the pathogen was detected in areas with both positive and negative values of PC2 and PC3 (**Figure S5**).

**Figure 4:**
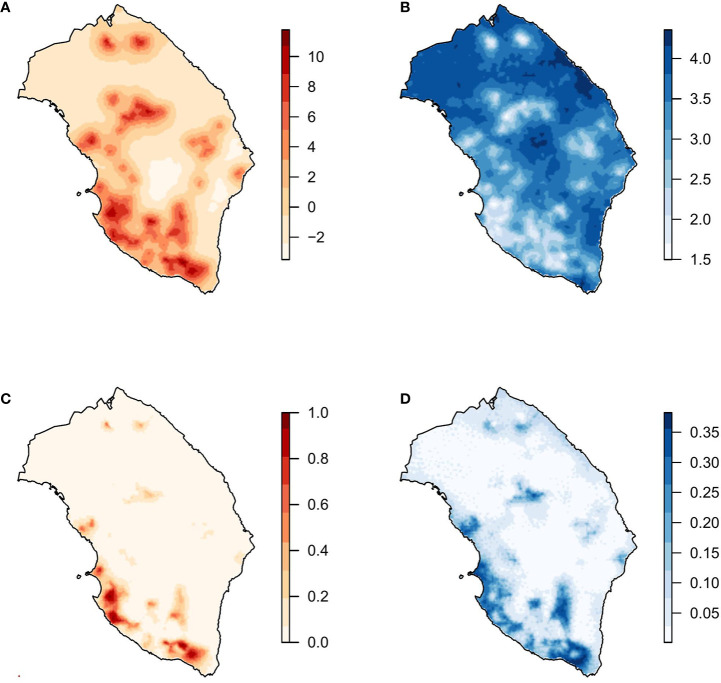
Model with principal components and spatial effect. **(A)** Mean and **(B)** standard deviation of the posterior distribution of the spatial effect. **(C)** Mean and **(D)** standard deviation of the posterior predictive distribution of the probability of *X. fastidiosa* presence in Lecce, Italy.

All model combinations (n = 16) with the three PCs and the spatial effect were fitted. The best model based on the WAIC criteria was the one including the three PCs and the spatial effect. Considering that WAIC values were virtually equivalent, the model including PC2, PC3, and the spatial effect was selected as the best one. Since the estimated parameters associated with PC2 and PC3 were negative in the model including the spatial effect (**Table 3**), higher values of these covariates were associated with a lower probability of the presence of *X. fastidiosa*. Nevertheless, the spatial component had the strongest effect on the model. Mean and standard deviation of the posterior distribution of the spatial effect were similar to those obtained with the model including climatic covariates, with higher values and lower variability in areas where the pathogen was detected (**Figure 4**). The predictive posterior distribution was also similar to that obtained with the model including climatic covariates. However, due to the effect of the PCs, in this case the highest probability of the presence of *X. fastidiosa* was concentrated on the Ionian coast and was lower in other areas of the province. Moreover, the standard deviation (uncertainty) of the predictive posterior distribution was higher in the areas with a higher probability.

**Table 3:** Mean, standard deviation (sd), quantiles (*Q*), and mode for the parameters and hyperparameters (ϕ and *σ_w_*) of the best model for the distribution of *X. fastidiosa* in Lecce, Italy, based on the second and third principal components (PC2 and PC3).

Parameters	Mean	sd	*Q*_0.025_	*Q*_0.5_	*Q*_0.975_	Mode
*β*_0_	-9.720	1.564	-13.316	-9.530	-7.203	-9.146
*PC*2	-1.139	0.543	-2.299	-1.110	-0.146	-1.056
*PC*3	-0.811	0.399	-1.628	-0.799	-0.056	-0.778
**Hyperparameters**	**Mean**	**sd**	***Q*_0.025_**	***Q*_0.5_**	***Q*_0.975_**	**Mode**
ϕ	5609.527	1112.976	3858.475	5455.545	8202.005	5132.501
*σ_w_*	4.837	0.753	3.527	4.778	6.480	4.662

β_0_ is the intercept and PC2 and PC3 represent the parameters of the second and third principal components, respectively. ϕ and σ_w_ are the range and variance of the spatial effect, respectively.

## Discussion

The climatic covariates presented low variability in both regions, probably due to the limited extent of the study areas. Despite this, the four categories defined by Purcell based on the minimum winter temperature (Anas et al., 2008) were all represented in Alicante. This was noteworthy, since it is not common to find all four categories in the same study area, and made it possible to infer whether the geographic distribution of the pathogen was somehow constrained by the low temperatures (Purcell, 1980; Lieth et al., 2011). Several studies have shown that successful *X. fastidiosa* infections (i.e., systemic host colonization) depend on certain factors like the temperature. For example, exposure of infected grapevines to low temperatures can effectively reduce or eliminate the pathogen, this phenomenon is known as “cold curing” (Purcell, 1980; Feil and Purcell, 2001). Actually, the climatic variables (temperature, precipitation) have a great influence on shaping the ecological conditions, which can (i) be more or less favorable for the insect vectors, in terms of population abundance, seasonal fluctuation, and attitude for dispersal and (ii) influence the abiotic stresses and consequently the severity of the symptoms associated with *X. fastidiosa* infections.

Given the results obtained in Alicante, *X. fastidiosa* subsp. *multiplex* was detected in similar proportions in all Purcell’s categories. This is in agreement with the known global distribution of this subspecies, which is present in warm climates, but also in areas characterized by cold winters such as Canada (Goodwin and Zhang, 1997). This is also in line with the results of Godefroid et al. (2019), suggesting that the subsp. *multiplex* may be more tolerant to cold temperatures. On the contrary, recent studies found a positive association with minimum winter temperatures in areas of France where *X. fastidiosa* subsp. *multiplex* is prevalent (Abboud et al., 2019; Martinetti and Soubeyrand, 2019). All this information together would suggested a greater environmental plasticity of subsp. *multiplex* compared to subsp. *fastidiosa*, and thus Purcell’s categories for *fastidiosa* would not be applicable to *multiplex*. Nevertheless, disease severity was not recorded in the dataset from Alicante, but only the presence/absence of *X. fastidiosa*. Our empirical observations in Alicante indicated that no major differences in almond leaf scorch severity were observed among Purcell’s four categories. In any case, formal quantification of disease severity in Alicante with a proper sample size and standard area diagrams would be needed to confirm this.

In Lecce, only two of Purcell’s categories were represented (i.e., severe and moderate). Thus, no conclusions could be drawn in relation to the potential effect of low winter temperature thresholds on the geographic distribution of *X. fastidiosa* subsp. *pauca* in this region. This subspecies is prevalent in southern Italy, including the study area in Lecce (Saponari et al., 2018), and it is widespread in Central and South America (Nunney et al., 2014a; Haelterman et al., 2015; Coletta-Filho et al., 2016). It was also detected in Provence-Alpes-Côte d’Azur (France) (DG SANTE, 2020) and the Balearic Islands (Spain) (EFSA, 2019). The climates in all these regions are characterized by mild winters, suggesting that *X. fastidiosa* subsp. *pauca* has a lower tolerance to cold temperatures. Nevertheless, data from experiments under controlled conditions are needed to support this hypothesis.

Multicollinearity refers to the non-independence between the covariates, which can lead to inaccurate estimation of the parameters of the model and a bias in the statistical inference, thus inducing an incorrect identification of the relevant covariates in the model (Dormann et al., 2013; Graham, 2003). Climatic covariates are typically correlated, as was our case. In this study, two different methods were used to minimize this problem. The first one was based on the pairwise correlation of the climatic variables, selecting only those with |*r*|<0.7. On the other hand, PCA was performed including all climatic variables. This is one of the most popular methods to reduce the number of covariates and avoid multicollinearity (Jolliffe, 2002; Dormann et al., 2013). Unlike the previous method based on pairwise correlations, PCA makes it possible to retain all the information provided by the set of variables through their linear combinations in PCs. Nevertheless, PCA has the disadvantage that the interpretation of the PCs linking the response variable and the covariates is not always straightforward. The zone near the coast in the study area in Alicante was characterized by high values of PC1 and low values of PC2 and PC3, meaning high values of the mean temperature of the coldest quarter and the precipitation seasonality, but low precipitation levels in the wettest month and in the mean diurnal range. In contrast, the lowest values of PC1 and the highest values of PC2 were located in a small central zone at a higher altitude, where samples were not available. On the other hand, in Lecce, the lowest values of PC1, meaning low mean diurnal range and high temperatures in the coldest month, were found on the Ionian coast, where most of the positive samples were concentrated.

In Alicante, the models showed that climate effects, included as climatic covariates or PCs, were not relevant. Therefore, no straightforward relationships could be established between the distribution of *X. fastidiosa* and temperature or precipitation in the study area. As indicated above, this may be due to the limited extent of the study area and the resulting relatively low variability of climatic variables. Nevertheless, the greater environmental plasticity of *X. fastidiosa* subsp. *multiplex* might have also played a role (EFSA, 2019). The best model based on the WAIC, LCPO and parsimony criteria included only the spatial effect. This implies that the areas close to positive findings of *X. fastidiosa* are more likely to be infested than those farther away. Our results also indicate that climatic factors are not likely to prevent the colonization of neighboring areas by *X. fastidiosa*. Therefore, control measures based on the reduction of inoculum and vector populations should be enforced to limit further disease spread.

Nevertheless, this spatial effect depends on the predefined neighborhood structure. In the case of Alicante, grid cells with a distance of ≤2.5 km between their centroids were considered neighbors. This distance was established as being the shortest at which all grid cells with a resolution of 1x1 km had at least one neighbor, considering that non-sampled grid cells were not considered in the analysis. This might represent a limitation of our spatial model, which could be improved by increasing the spatial resolution of the dataset and defining alternative neighborhood structures based on actual disease spread distances.

The ability of several insect species to transmit *X. fastidiosa*, like *H. vitripennis* in southern California (Almeida and Purcell, 2003) or *P. spumarius* in Italy (Saponari et al., 2014; Cornara et al., 2017) is well documented. Daugherty and Almeida (2009) studied the ecology of two vectors of PD, *H. vitripennis* and *Graphocephala atropunctata* (Signoret) (Hemiptera: Cicadellidae), which are prevalent in coastal areas of California. Vector abundance together with the duration of the acquisition and inoculation periods greatly influenced the transmission efficiency of *X. fastidiosa* by insect species. Nevertheless, from a spatial epidemiology perspective, studies providing data on the actual distances of vector dispersal and disease spread are needed, especially for those present in Europe for which quantitative information is rather uncertain.

The models for the study area in Lecce showed that some climatic covariates could be related to the distribution of *X. fastidiosa* subsp. *pauca*. In particular, the mean diurnal range (*bio2*) and the mean temperature of the wettest quarter (*bio8*) were negatively related to the presence of the pathogen. That is, areas with higher variation in daily temperature and higher temperatures during the wettest months would have a lower probability of *X. fastidiosa* presence. Likewise, effects were observed with PC2 and PC3 in the model with PCA. In PC2, climatic covariates related to precipitation in the wettest month and the coldest quarter (*bio13* and bio19) made the greatest contribution, while PC3 was mainly defined by the *ADD* over 15°C from April to October. Both PC2 and PC3 had negative coefficients in the model, indicating a lower probability of presence in areas with more precipitation in winter and higher temperatures during the vegetative growth period of the olive tree.

From these results, it can be speculated that wet winters and hot summers in Lecce would be detrimental to vector activity and/or bacterial multiplication in the host plants. Nevertheless, given the environmental homogeneity found in the study area, the results of the model in terms of climatic covariates were inconclusive. The survey strategy was not uniform across the study area and most of the positive findings were concentrated around the first location of *X. fastidiosa* near Gallipoli (Martelli et al., 2016). Consequently, the heterogeneous distribution of the samples could be confounded with the climatic covariates.

Previous studies estimated the potential spread of *X. fastidiosa* in Europe, in order to implement control strategies and assess potential impacts. These studies used different methodologies like network analysis (Strona et al., 2017; Strona et al., 2020), spatially explicit process-based models (White et al., 2017; EFSA, 2019), compartmental Susceptible-Infected-Removed (SIR) Bayesian models (Soubeyrand et al., 2018), or a coupled reaction-diffusion-absorption model, considering the spread *via* insects and transportation of plants (Abboud et al., 2019). Nevertheless, uncertainties on the actual vector dispersal and disease spread distances in Europe as well as on the human-assisted dispersal component limit the predictive capacity of the models. In our case, *X. fastidiosa* datasets from Alicante and Lecce were analyzed with different methodologies because they were actually two different types of spatial data. In Alicante, the georeferenced samples were presented on a discrete space (lattice data) so that the spacial dependence was incorporated through an ICAR structure, which is a particular case of GMRF. In this way, the Besag, York, and Mollie model was implemented (Besag et al., 1991) using the reparameterization proposed by Simpson et al. (2017), which includes a standardized spatial effect. In the case of Lecce, locations were considered in a continuous space (geostatistical data), so the SPDE was used (Lindgren et al., 2011), where the GF is represented through Matérn covariance as GMRF in order to use the INLA methodology.

The effect of spatial relationships in SDMs cannot be ignored, as is clearly illustrated by our study, where the spatial effect explained virtually all the variability found in the distribution of *X. fastidiosa* in both regions. Therefore, in our particular case studies, models which did not consider spatial autocorrelation could result in erroneous relationships between some covariates and the presence/absence of the pathogen. The use of Bayesian hierarchical models allowed a straightforward incorporation of the spatial effect, which would indeed be challenging from a frequentist approach. This methodology allowed sources of variability and unobserved uncertainty to be incorporated in a convenient way. Furthermore, INLA has proven to be a computationally efficient methodology to implement complex Bayesian hierarchical models including spatial autocorrelation.

In both study areas, the spatial component had a strong effect in the models regardless of the climatic variables. This substantial contribution of the spatial effect in the models might indicate that the current extent of *X. fastidiosa* in the study regions had arisen from a single outbreak in each zone or several nearby outbreaks that coalesced. Nevertheless, actual disease spread rates based on time-series data would be needed to confirm this hypothesis. In the case of Lecce, data were indeed available from 2013 to 2018, but with different surveillance strategies in each campaign, to comply with the updates of Decision (EU) 2015/789. This temporal and spatial heterogeneity in surveillance constrained the information that can be derived from the dataset, and only data from the first campaign and related to one province met the requirements to be used in model fitting. From an epidemiological modeling perspective, a recommendation for risk managers would be to perform additional surveillance programs, complementary to those established by Decision (EU) 2015/789, to gather more informative epidemiological data and draw sound conclusions on the spatio-temporal scale of disease spread. Finally, the spatial models developed here may assist risk managers in designing more efficient surveillance strategies, where inspection and sampling efforts would be adjusted considering the probability of *X. fastidiosa* presence.

## Data Availability Statement

All datasets generated for this study are included in the https://bitbucket.org/mcendoya/xylella_alicante_lecce.

## Author Contributions

MC analyzed the data and wrote the original draft. JM-M contributed with the statistical analysis. VD, AF, and MS provided the original data. MC, DC, AL-Q, and AV contributed conception and design of the study. All authors contributed to the article and approved the submitted version.

## Funding

The present work has received funding from European Union’s Horizon 2020 research and innovation program under grant agreement no. 727987 (XF-ACTORS, “Xylella Fastidiosa Active Containment Through a multidisciplinary-Oriented Research Strategy”), grant E-RTA 2017-00004-C06-01 (FEDER INIA AEI-MCIU and Organización Interprofesional del Aceite de Oliva Español), grants PID2019-106341GB-I100 MCI and TEC2016-81900-REDT (FEDER AEI-MCIU), and Basque Government BERC 2018-2021 program AEI-MCIU BCAM Severo Ochoa accreditation SEV-2017-0718. MC held an IVIA grant partially funded by the European Social Fund.

## Conflict of Interest

VD and AF are plant health officers of the competent authority [Regulation (EU) 2016/2031] in Comunitat Valenciana, Spain.

The remaining authors declare that the research was conducted in the absence of any commercial or financial relationships that could be construed as a potential conflict of interest.

The reviewer FM declared a past co-authorship with several of the authors AL-Q and DC to the handling editor.
