# Supplementary material for: Spatial Bayesian Modeling Applied to the Surveys of Xylella fastidiosa in Alicante (Spain) and Apulia (Italy)
Source: Front Plant Sci. 2020 Aug 14;11:1204. doi: 10.3389/fpls.2020.01204 (PMC7456931; doi:10.3389/fpls.2020.01204)
Supplement: Supplementary file 1 [file DataSheet_1.pdf]

frontiers

Supplementary Material

1 SUPPLEMENTARY TABLES AND FIGURES

1.1 Figures

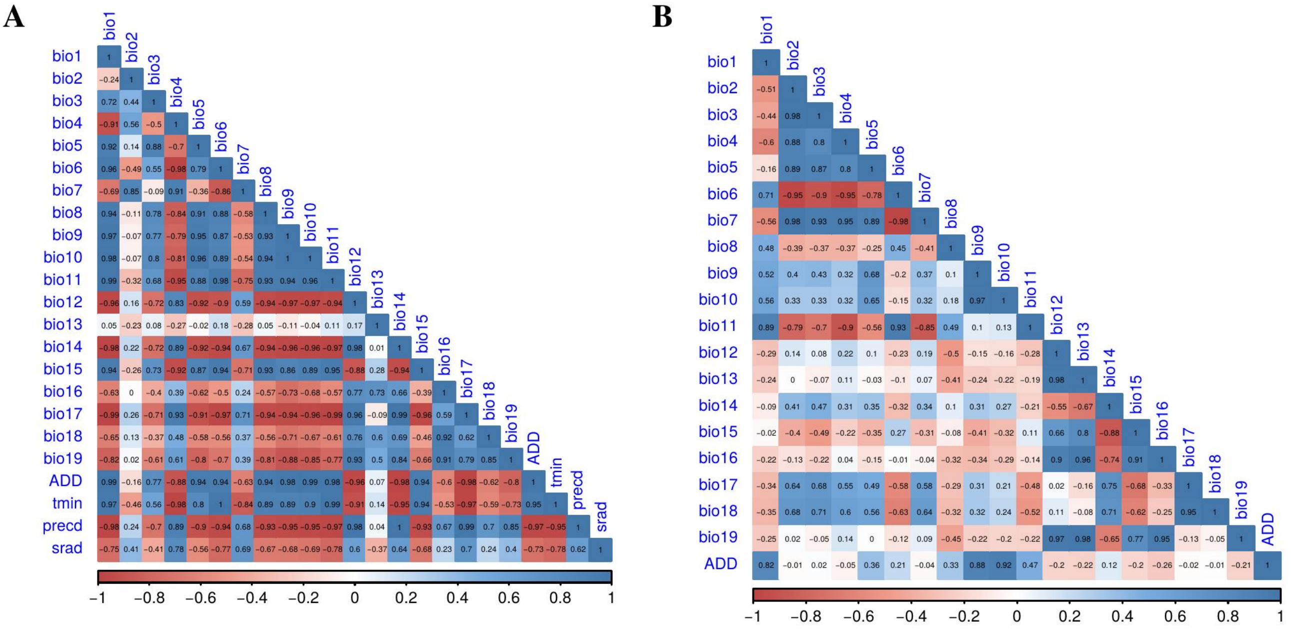

Figure S1: Correlation matrix. (**A**) Pearson correlation coefficient for bioclimatic variables (*bio*), accumulated degree-days over 15 °C from February to October (*ADD*), average minimum temperature in winter (*tmin*), average precipitation during the dry season (*precd*) and solar radiation (*srad*) in Alicante, Spain; (**B**) Pearson correlation coefficient for bioclimatic variables (*bio*) and accumulated degree-days over 15 °C from April to October (*ADD*) in Lecce, Italy.

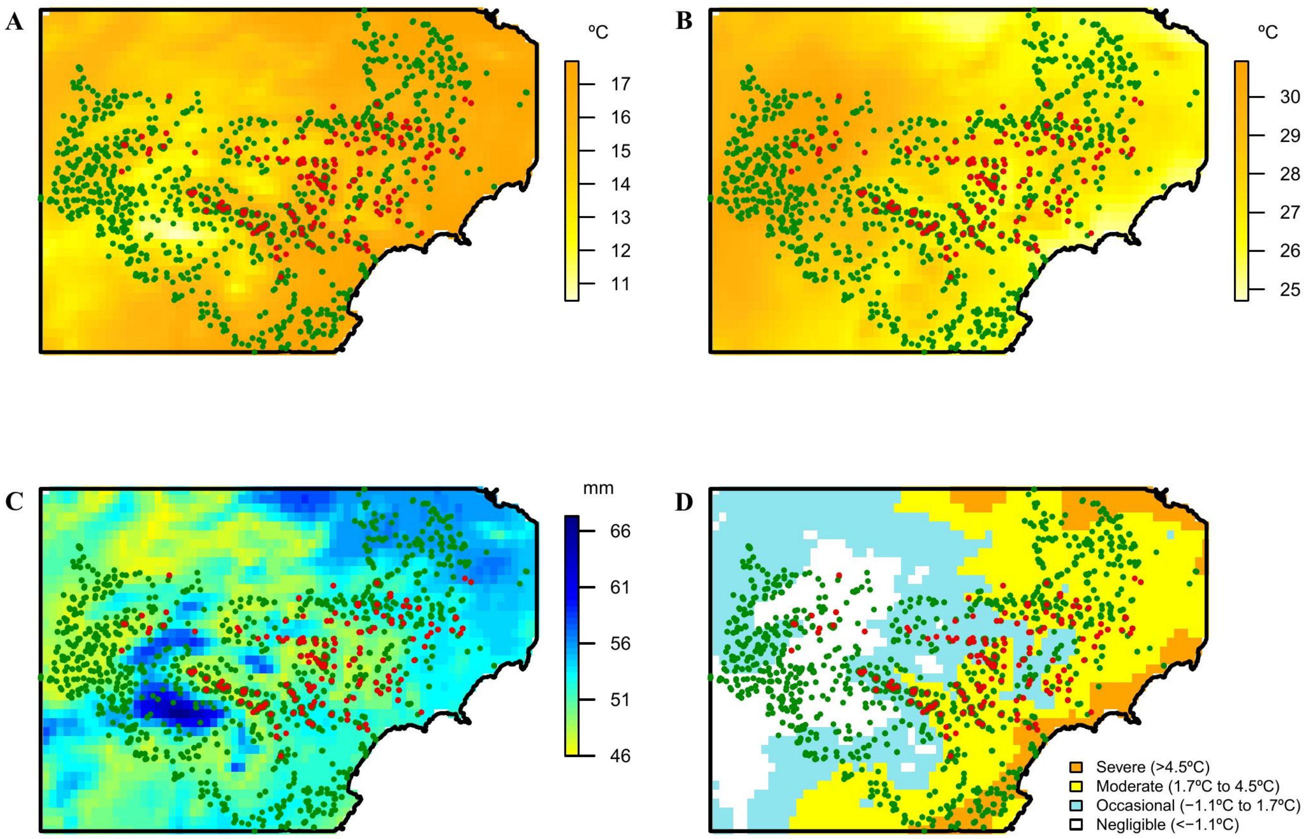

Figure S2: Geographical distribution of (●) presence and (●) absence of *Xylella fastidiosa* over climatic covariates in the demarcated area in Alicante, Spain; **(A)** annual mean temperature (*bio1*), **(B)** temperature annual range (*bio7*), **(C)** precipitation of the wettest month (*bio13*), and **(D)** Purcell's categories based on minimum winter temperature (Anas et al., 2008).

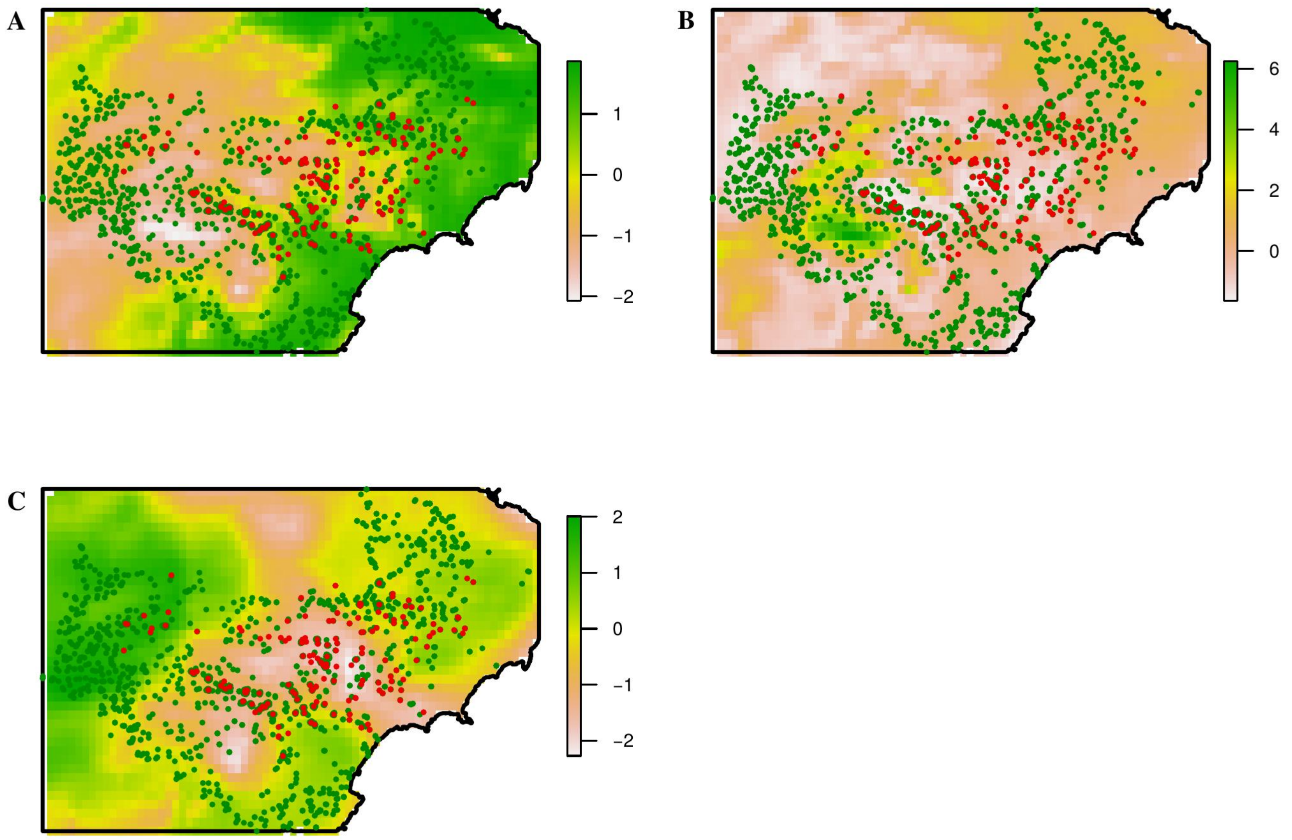

Figure S3: Geographical distribution of (●) presence and (●) absence of *Xylella fastidiosa* in the demarcated area in Alicante, Spain, over the principal components, **(A)** PC1, **(B)** PC2 and **(C)** PC3.

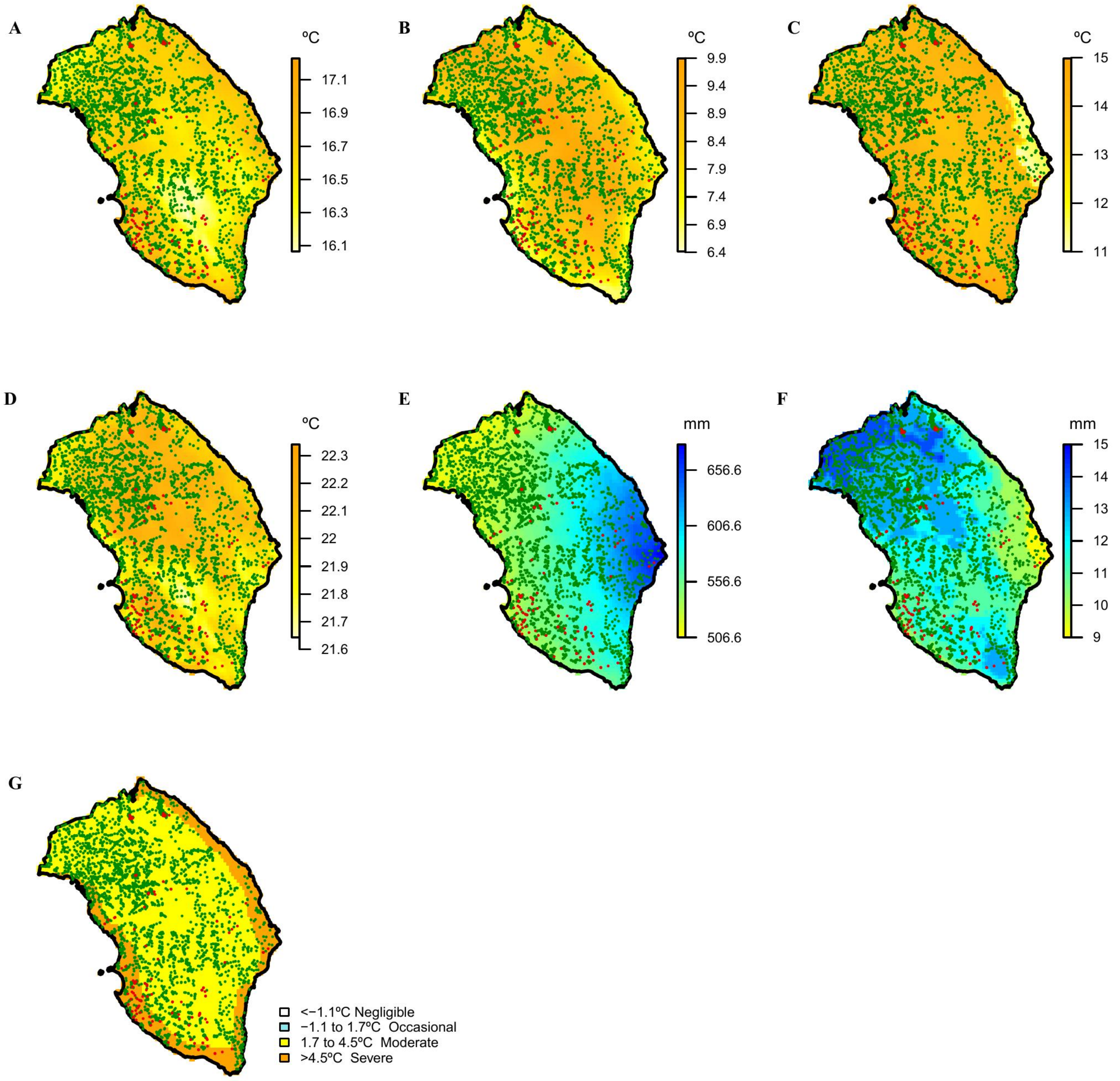

Figure S4: Geographical distribution of (●) presence and (●) absence of *Xylella fastidiosa* over climatic covariates in Lecce, Italy **(A)** annual mean temperature *bio1*, **(B)** mean diurnal range *bio2*, **(C)** mean temperature of the wettest quarter *bio8*, **(D)** mean temperature of the driest quarter *bio9*, **(E)** annual precipitation *bio12*, **(F)** precipitation of the driest month *bio14*, and **(G)** Purcell's categories based on minimum winter temperature (Anas et al., 2008).

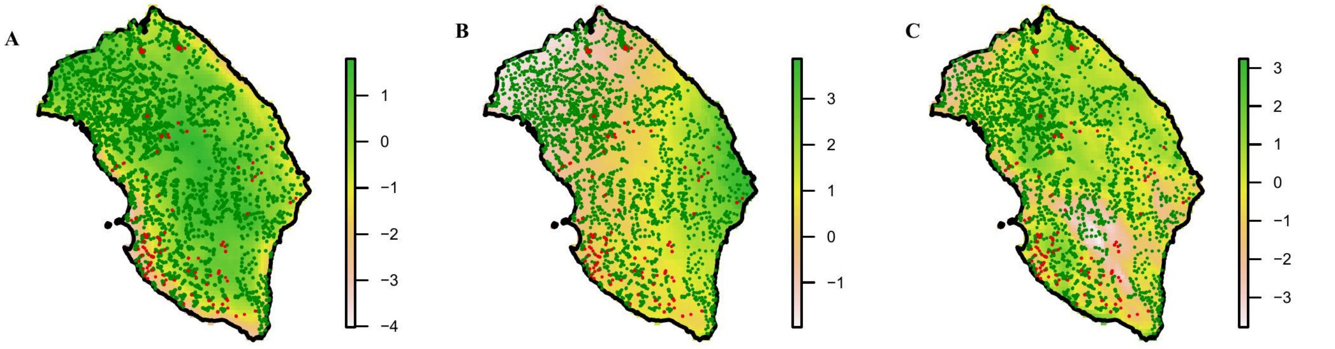

Figure S5: Geographical distribution of (●) presence and (●) absence of *Xylella fastidiosa* in Lecce, Italy, over the principal components **(A)** PC1, **(B)** PC2, and **(C)** PC3.

1.2 Tables

Table S1: Best models for the distribution of *Xylella fastidiosa* in the demarcated area in Alicante, Spain.

	Model[a]	WAIC[b]	LCPO[c]
Models with climatic covariates			
1.	$\beta_0 + \boldsymbol{v}$	617.627	1.638
2.	$\beta_0 + bio7 + \boldsymbol{v}$	617.922	1.650
3.	$\beta_0 + bio1 + \boldsymbol{v}$	618.027	1.639
4.	$\beta_0 + bio1 + bio13 + \boldsymbol{v}$	620.013	1.653
5.	$\beta_0 + bio13 + \boldsymbol{v}$	620.293	1.641
6.	$\beta_0 + bio1 + bio7 + \boldsymbol{v}$	620.530	1.636
7.	$\beta_0 + bio7 + bio13 + \boldsymbol{v}$	620.620	1.661
8.	$\beta_0 + PRC + \boldsymbol{v}$	621.386	1.672
9.	$\beta_0 + bio1 + bio7 + bio13 + \boldsymbol{v}$	622.304	1.641
10.	$\beta_0 + bio1 + PRC + \boldsymbol{v}$	622.520	1.675
Models with principal components (*PC*)			
1.	$\beta_0 + \boldsymbol{v}$	617.627	1.638
2.	$\beta_0 + PC2 + \boldsymbol{v}$	618.887	1.656
3.	$\beta_0 + PC1 + \boldsymbol{v}$	619.558	1.635
4.	$\beta_0 + PC1 + PC2 + \boldsymbol{v}$	620.005	1.656
5.	$\beta_0 + PRC + \boldsymbol{v}$	621.399	1.672
6.	$\beta_0 + PC2 + PRC + \boldsymbol{v}$	622.634	1.689
7.	$\beta_0 + PC2 + PC3 + \boldsymbol{v}$	623.140	1.599
8.	$\beta_0 + PC1 + PC2 + PC3 + \boldsymbol{v}$	623.171	1.634
9.	$\beta_0 + PC1 + PC3 + \boldsymbol{v}$	623.364	1.608
10.	$\beta_0 + PC3 + \boldsymbol{v}$	623.530	1.584

[a] Intercept (β_0), annual mean temperature (*bio1*), temperature annual range (*bio7*), precipitation of the wettest month (*bio13*), first principal component ($PC1$), second principal component ($PC2$), third principal component ($PC3$), Purcell's risk categories (PRC) and spatial effect ($\boldsymbol{v}$).

[b] Watanabe Akaike information criterion (WAIC).

[c] Logarithmic score of conditional predictive ordinate (LCPO).

Table S2: Principal component analysis (PCA) loadings for climatic variables in the demarcated area in Alicante, Spain, and Lecce, Italy.

	Alicante			Lecce		
Climatic covariates	PC1	PC2	PC3	PC1	PC2	PC3
bio1	0.963	-0.260	0	-0.636	-0.152	0.731
bio2	-0.236	0	0.967	0.955	0	0.138
bio3	0.750	0	0.633	0.923	-0.117	0.160
bio4	-0.926	0	0.347	0.919	0.102	0
bio5	0.888	-0.267	0.343	0.804	0	0.506
bio6	0.947	-0.146	-0.277	-0.964	0	0
bio8	0.934	-0.217	0.107	-0.502	-0.388	0.236
bio9	0.900	-0.388	0.119	0.286	-0.170	0.930
bio10	0.930	-0.320	0.134	0.216	-0.130	0.952
bio11	0.972	-0.202	-0.107	-0.870	-0.147	0.350
bio12	-0.882	0.460	0	0.228	0.922	0
bio13	0.297	0.936	-0.110	0	0.969	0
bio14	-0.937	0.322	0	0.458	-0.782	0
bio15	0.976	0	0	-0.397	0.869	-0.148
bio16	-0.404	0.902	0	0	0.967	-0.16
bio17	-0.967	0.222	0	0.755	-0.327	0
bio18	-0.445	0.836	0	0.796	-0.240	0
bio19	-0.659	0.721	0	0.101	0.959	0
ADD	0.968	-0.228	0	-0.141	-0.121	0.973
tmin	0.949	-0.179	-0.257	-	-	-
precd	-0.925	0.346	0	-	-	-
srad	-0.789	-0.147	0.258	-	-	-
% variability	69.7	18.2	8.5	38.9	28.7	20.1

Table S3: Best models for the distribution of *Xylella fastidiosa* in Lecce, Italy.

	Model[a]	WAIC[b]	LCPO[c]
Models with climatic covariates			
1.	$\beta_0 + bio1 + bio2 + bio9 + \boldsymbol{w}$	1091.485	0.131
2.	$\beta_0 + bio2 + bio8 + bio9 + bio12 + \boldsymbol{w}$	1095.184	0.132
3.	$\beta_0 + bio2 + bio8 + bio12 + \boldsymbol{w}$	1095.307	0.132
4.	$\beta_0 + bio1 + bio2 + bio8 + bio12 + \boldsymbol{w}$	1095.709	0.132
5.	$\beta_0 + bio2 + bio8 + bio9 + \boldsymbol{w}$	1095.959	0.132
6.	$\beta_0 + bio2 + bio8 + bio9 + bio12 + bio14 + \boldsymbol{w}$	1096.218	0.132
7.	$\beta_0 + bio2 + bio8 + \boldsymbol{w}$	1096.343	0.132
8.	$\beta_0 + bio2 + bio8 + bio12 + bio14 + \boldsymbol{w}$	1096.348	0.132
9.	$\beta_0 + bio2 + bio12 + \boldsymbol{w}$	1096.362	0.132
10.	$\beta_0 + bio1 + bio2 + bio8 + \boldsymbol{w}$	1096.692	0.132
Models with principal components (*PC*)			
1.	$\beta_0 + PC1 + PC2 + PC3 + \boldsymbol{w}$	1078.769	0.130
2.	$\beta_0 + PC1 + PC3 + \boldsymbol{w}$	1079.789	0.130
3.	$\beta_0 + PC2 + PC3 + \boldsymbol{w}$	1080.430	0.130
4.	$\beta_0 + PC1 + PC2 + \boldsymbol{w}$	1081.277	0.130
5.	$\beta_0 + PC3 + \boldsymbol{w}$	1081.607	0.133
6.	$\beta_0 + PC1 + \boldsymbol{w}$	1081.974	0.133
7.	$\beta_0 + PC2 + \boldsymbol{w}$	1082.573	0.133
8.	$\beta_0 + \boldsymbol{w}$	1083.363	0.133
9.	$\beta_0 + PC1 + PC2$	1711.042	0.133
10.	$\beta_0 + PC1 + PC2 + PC3$	1712.478	0.133

[a] Intercept (β_0), annual mean temperature (*bio1*), mean diurnal range (*bio2*), mean temperature of the wettest quarter (*bio8*), mean temperature of the driest quarter (*bio9*), annual precipitation (*bio12*), precipitation of the driest month (*bio14*), first principal component ($PC1$), second principal component ($PC2$), third principal component ($PC3$), and spatial effect ($\boldsymbol{w}$).

[b] Watanabe Akaike information criterion (WAIC).

[c] Logarithmic score of conditional predictive ordinate (LCPO).

REFERENCES

Anas, O., Harrison, U. J., and Brannen, P. M. (2008). The effect of warming winter temperatures on the severity of Pierce’s disease in the Appalachian mountains and Piedmont of the southeastern United States. *Plant Health Progress* 9, 13. doi:10.1094/PHP-2008-0718-01-RS
